# SARS-CoV-2 Seroprevalence among a Southern U.S. Population Indicates Limited Asymptomatic Spread under Physical Distancing Measures

**DOI:** 10.1128/mBio.02426-20

**Published:** 2020-09-29

**Authors:** Amir Barzin, John L. Schmitz, Samuel Rosin, Rameet Sirpal, Martha Almond, Carole Robinette, Samantha Wells, Michael Hudgens, Andrew Olshan, Stephanie Deen, Patrick Krejci, Eugenia Quackenbush, Kevin Chronowski, Caleb Cornaby, Janette Goins, Linda Butler, Julia Aucoin, Kim Boyer, Janet Faulk, Devena Alston-Johnson, Cristen Page, Yijun Zhou, Lynne Fiscus, Blossom Damania, Dirk P. Dittmer, David B. Peden

**Affiliations:** a The University of North Carolina at Chapel Hill School of Medicine, Department of Family Medicine, Chapel Hill, North Carolina, USA; b The University of North Carolina at Chapel Hill School of Medicine, Department of Pathology and Laboratory Medicine and UNC Hospitals McLendon Clinical Laboratories, Chapel Hill, North Carolina, USA; c The University of North Carolina at Chapel Hill Gillings School of Global Public Health, Department of Biostatistics, Chapel Hill, North Carolina, USA; d UNC Physicians Network, Chapel Hill, North Carolina, USA; e The University of North Carolina at Chapel Hill School of Medicine, Center for Environmental Medicine, Asthma and Lung Biology, Chapel Hill, North Carolina, USA; f The University of North Carolina at Chapel Hill Gillings School of Global Public Health, Department of Epidemiology, Chapel Hill, North Carolina, USA; g UNC Health Information Services Division, Chapel Hill, North Carolina, USA; h The University of North Carolina at Chapel Hill School of Medicine, Department of Emergency Medicine, Chapel Hill, North Carolina, USA; i The University of North Carolina at Chapel Hill, North Carolina Translational and Clinical Sciences Institute, Chapel Hill, North Carolina, USA; j UNC REX Healthcare, Raleigh, North Carolina, USA; k UNC Health Nash General Hospital, Rocky Mount, North Carolina, USA; l The University of North Carolina at Chapel Hill School of Medicine, Department of Microbiology and Immunology, Chapel Hill, North Carolina, USA; m The University of North Carolina at Chapel Hill School of Medicine, Lineberger Comprehensive Cancer Center, Chapel Hill, North Carolina, USA; n The University of North Carolina at Chapel Hill School of Medicine, Department of Pediatrics, Chapel Hill, North Carolina, USA; St. Jude Children's Research Hospital

**Keywords:** COVID-19, SARS-CoV-2, antibody, coronavirus, seroprevalence

## Abstract

Characterizing the asymptomatic spread of SARS-CoV-2 is important for understanding the COVID-19 pandemic. This study was aimed at determining asymptomatic spread of SARS-CoV-2 in a suburban, Southern U.S. population during a period of state restrictions and physical distancing mandates. This is one of the first published seroprevalence studies from North Carolina and included multicenter, primary care, and emergency care facilities serving a low-density, suburban and rural population since description of the North Carolina state index case introducing the SARS-CoV-2 respiratory pathogen to this population. To estimate point seroprevalence of SARS-CoV-2 among asymptomatic individuals over time, two cohort studies were examined. The first cohort study, named ScreenNC, was comprised of outpatient clinics, and the second cohort study, named ScreenNC2, was comprised of inpatients unrelated to COVID-19. Asymptomatic infection by SARS-CoV-2 (with no clinical symptoms) was examined using an Emergency Use Authorization (EUA)-approved antibody test (Abbott) for the presence of SARS-CoV-2 IgG. This assay as performed under CLIA had a reported specificity/sensitivity of 100%/99.6%. ScreenNC identified 24 out of 2,973 (0.8%) positive individuals among asymptomatic participants accessing health care during 28 April to 19 June 2020, which was increasing over time. A separate cohort, ScreenNC2, sampled from 3 March to 4 June 2020, identified 10 out of 1,449 (0.7%) positive participants.

## Abstract (precis)

This study suggests limited but accelerating asymptomatic spread of SARS-CoV-2. Asymptomatic infections, like symptomatic infections, disproportionately affected vulnerable communities in this population, and seroprevalence was higher in African American participants than in White participants. The low, overall prevalence may reflect the success of shelter-in-place mandates at the time this study was performed and of maintaining effective physical distancing practices among suburban populations. Under these public health measures and aggressive case finding, outbreak clusters did not spread into the general population.

## INTRODUCTION

Acute virus infections typically induce a vigorous antibody response. The overwhelming majority of COVID-19 patients also produce antibodies, initially of the IgM isotype and shortly thereafter of the IgG isotype. Seroconversion rates approach 100% at 3 weeks after symptoms appear and persist for several months (1–4). The composition, quality, median and maximal duration of the IgG response are unclear as the earliest cases occurred less than 7 months ago (5). Both presymptomatic and asymptomatic transmission events of SARS-CoV-2 have been reported (6–11). It is difficult, however, to define asymptomatic transmission *a priori*, as the full spectrum of SARS-CoV-2-associated clinical symptoms is still evolving (12, 13). The degree to which asymptomatic transmission contributes to population seroprevalence and eventual herd immunity is unknown and the subject of intense debate.

Determining the extent to which SARS-CoV-2 can be transmitted asymptomatically shapes COVID-19 disease modeling and informs public health interventions. Asymptomatic transmission represents a key biological property of this virus. Early serology studies for SARS-CoV-2 were conducted in densely populated, urban settings, such as Wuhan, China; Bergamo, Italy; or Boston, New York, Los Angeles, or Seattle in the United States (14, 15). These studies focused on patients who recovered after severe clinical disease (4). They were conducted at hot spots of transmission, in urban settings with multiple independent introductory events and extended opportunities for rapid spread, such as multiple modes of public transport and international travel (16).

North Carolina (NC), in contrast, represents a largely suburban and rural population with limited public transport. The first case of COVID-19 was reported on 2 March 2020. As in other locales, symptomatic cases were concentrated among vulnerable populations, such as residents of retirement homes, prison inmates, and workers in meat-processing plants. “Stay-at-home” directives were implemented 30 March to 8 May 2020. Statewide, all schools were ordered to close 14 March and did not reopen during the study period. As of 19 June, NC reported 49,840 cases and 1,197 deaths (NC Department of Health and Human Services [DHHS]) for a population of 10,488,084 people (2019 census), based primarily on nucleic acid testing (NAT) of suspected cases. Thus, the fraction of infected persons based on case counts for this area and calendar time using viral NAT is 0.5%.

## RESULTS

This study evaluated two cohorts of asymptomatic patients. ScreenNC enrolled 2,973 asymptomatic participants across the University of North Carolina Health System (UNC Health), representing 267 different ZIP codes. These individuals sought medical care unrelated to COVID-19. Individuals with known COVID-19 symptoms, at screening or in their recent past, were excluded. ScreenNC2 tested 1,449 sera from patients in clinical care not related to COVID-19 or recruited to the study. No patient was tested more than once. ScreenNC enrolled 28 April to 19 June 2020, and ScreenNC2 sampled patients 3 March to 4 June 2020.

Table 1 shows the demographic composition of the ScreenNC cohort compared to patients accessing UNC Health sites during the same time period in 2020 as well as in 2019, i.e., pre-COVID-19. Table 2 shows the age distribution. There were no differences between overall patient populations accessing UNC Health in 2020 compared to 2019. When comparing the ScreenNC population and patient populations accessing UNC Health in 2020, there were some notable differences. ScreenNC enrolled fewer 80+-year-old patients who accessed care during the same calendar period (2.6% versus 7.4%), slightly more females (65.8% versus 63.6%), fewer Black or African American participants (13.3% versus 23.3%, *P* ≤ 0.001 by Fisher exact test), and fewer Hispanic or Latino participants than the comparator UNC Health population (2.6% versus 4.9%, *P* ≤ 0.001 by Fisher exact test) during the same calendar period. These differences were used in a model of adjusted seroprevalence.

**TABLE 1:** Cohort demographics comparing the study cohort to patient populations accessing the same UNC Health facilities during the same calendar period in 2019 and 2020

	ScreenNC (*n* = 2,973)	UNC 2019 (*n*= 31,095)	UNC 2020 (*n*= 21,901)
Sex			
Female	1,955 (65.8%)	19,623 (63.1%)	13,926 (63.6%)
Male	1,018 (34.2%)	11,472 (36.9%)	7,975 (36.4%)

Race			
Asian	67 (2.3%)	661 (2.1%)	460 (2.1%)
Black or African American	395 (13.3%)	7,121 (22.9%)	5,109 (23.3%)
Other	141 (4.7%)	1,652 (5.3%)	1,256 (5.7%)
Patient refused or unknown	170 (5.7%)	587 (1.9%)	543 (2.5%)
White or Caucasian	2,200 (74.0%)	21,074 (67.8%)	14,533 (66.4%)

Ethnic group			
Hispanic or Latino	76 (2.6%)	1,371 (4.4%)	1,078 (4.9%)
Not Hispanic or Latino	2,637 (88.7%)	28,727 (92.4%)	19,979 (91.2%)
Patient refused	8 (0.3%)	88 (0.3%)	59 (0.3%)
Unknown	252 (8.5%)	909 (2.9%)	785 (3.6%)

**TABLE 2:** Age characteristics

Age group (yr)	SNC filtered age 20+ (*n* = 2,937)	UNC 2019 (*n*= 31,095)	UNC 2020 (*n*= 21,901)
20–29^*a*^	342 (11.6%)	2,541 (8.2%)	2,060 (9.4%)
30–39	599 (20.4%)	3,330 (10.7%)	2,763 (12.6%)
40–49	518 (17.6%)	4,337 (13.9%)	3,382 (15.4%)
50–59	602 (20.5%)	5,560 (17.9%)	4,200 (19.2%)
60–69	489 (16.6%)	6,606 (21.2%)	4,548 (20.8%)
70–79	310 (10.6%)	5,777 (18.6%)	3,325 (15.2%)
80–plus	77 (2.6%)	2,944 (9.5%)	1,623 (7.4%)

a ScreenNC (SNC) was restricted to participants older than 18 years, and no separate hospital population data for 18- and 19-year-old patients were available.

SARS-CoV-2 IgG antibody was detected using the Abbott SARS-CoV-2 IgG assay on the Architect platform under Emergency Use Authorization (EUA). Index values of ≥1.4 were considered positive. This assay has a manufacturer reported analytical sensitivity of 100% and specificity of 99.6%. Independent studies in the U.S. population report sensitivities/specificities of 100%/99.9% (17), 92.9%/99.6% (18), 99.0%/99.8% (24), and 100%/99.6% (25), respectively. On-site validation (*n* = 317) established a sensitivity of 100% and a specificity of 98.9% at 3 weeks after symptom onset. Intra-assay precision (% coefficient of variation [CV%]) was 1.1%, and interassay CV% was 0.92%. Thus, in-house performance was comparable to the manufacturer’s specification and to other studies in the United States.

**FIG 1:**
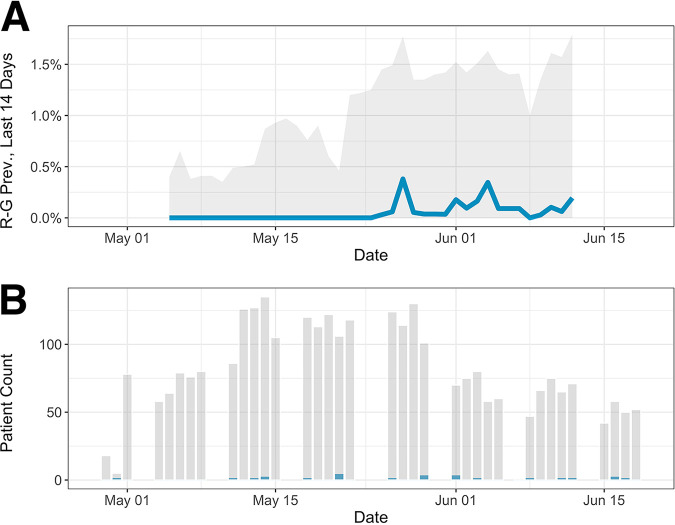
Sample seroprevalence over time. (A) The vertical axis shows the 2-week moving average of Rogan-Gladen-adjusted seroprevalence (blue) with 95% bootstrap confidence intervals (gray). This averaging includes weeks where no positive tests were recorded, yielding to a temporary decline of the prevalence estimate. (B) Daily patient count (gray) and positive case counts (blue). The calendar date is shown on the horizontal axis.

ScreenNC identified 24 out of 2,973 (0.8%) positive participants, and ScreenNC2 identified 10 out of 1,449 (0.7%) positive participants. Black or African American participants had twice the unadjusted seropositivity rate of Whites (1.5% versus 0.7%). The analysis of other demographic factors was not possible due to the small number of positive cases. The unadjusted seroprevalence remained constant over time for the ScreenNC2 population but showed an upward, although imprecise, trend for the larger ScreenNC cohort (Fig. 1 and Table 3).

**TABLE 3:** Raw percent seroprevalence over time

Week (mo/day/yr)	ScreenNC	ScreenNC2
03/02/2020	N/A^*a*^	0.5%
03/30/2020	N/A	0.0%
04/06/2020	N/A	2.5%
04/13/2020	N/A	0.0%
04/20/2020	N/A	0.5%
04/27/2020	1.0%	2.6%
05/04/2020	0.0%	0.8%
05/11/2020	0.7%	0.0%
05/18/2020	0.9%	0.0%
05/25/2020	0.9%	N/A
06/01/2020	1.2%	0.0%
06/08/2020	0.9%	N/A
06/15/2020	1.4%	N/A

a N/A, not available.

The counts were adjusted for assay characteristics using the method of Rogan and Gladen (19) and cohort characteristics using direct standardization to yield a population point estimate of 0.0% (95% confidence interval [95% CI] of 0.0% to 0.9%, with *n* = 10,000 bootstraps) for the ScreenNC cohort. For ScreenNC2, demographic data were not available. In sum, the seroprevalence as estimated by antibody testing among asymptomatic participants was similar to the 0.5% fraction of infected persons based on case counts for this area and calendar time using viral NAT.

## DISCUSSION

This study identifies a very limited seroprevalence of SARS-CoV-2 among asymptomatic individuals accessing the UNC Health system. There was a suggestion of accelerating asymptomatic spread of SARS-CoV-2 during the study period and cohort, i.e., the transmission among persons who never subjectively felt ill. The seroprevalence of less than 1% was lower than estimates from earlier studies, but in line with recent studies using high-accuracy tests (15, 17). The seroprevalence for this low-density community was lower than reported in a large study of convenience samples that focused around “hot spot” cities and did not explicitly exclude persons with past symptomatic infections (20). This result may reflect the success of shelter-in-place state mandates and maintaining effective physical distancing among suburban populations during the time frame of our study. It may also reflect the low population density and delayed introduction of the virus compared to population centers and travel nodes. Under these circumstances, early outbreak clusters did not expand far into asymptomatic patients, which indicates that continued viral NAT and aggressive case finding can curb SARS-CoV-2 spread.

The data suggest that members of the Black community are disproportionately affected by the COVID-19 epidemic, here as demonstrated by asymptomatic acquisition of infection and in other studies as demonstrated by increased incidence of symptomatic cases and deaths. Significantly fewer members of the Black and Latinx communities participated in this study than accessed care in the same calendar time, which may have led to biased enrollment.

There are many possible explanations for the variation in population seroprevalence among different preliminary reports and published studies (14, 17). Our study populations were derived from individuals accessing health care clinics and may not provide a population-representative study sample. It is very challenging to conduct random population sampling during a public health emergency when “stay-at-home” orders are in place. Prevalence of transmission, patterns of behavior, and characteristics of the sample population were time varying during the sample period. Logistical constraints, such as lack of personal protective equipment and the primacy of clinical care, limited study access. In NC, only persons with a valid medical reason and “essential workers” were exempted from the “stay-at-home” ordinance, and only those participated in the study. The degree to which underlying health conditions impact analytical assay sensitivity is currently unknown; for instance, ScreenNC2 included patients with immune suppression and thus likely impairments in their ability to generate high-titer antibodies.

Defining “asymptomatic” is a challenge and a limitation, as it is based on self-reported clinical symptoms of infection, the exact definition of which continues to evolve. Both symptom severity and awareness may differ among different communities. Third, there exists a wide variety of antibody tests, each with differing performance characteristics (21, 22). These serological tests are validated on symptomatic, hospitalized patients, not asymptomatic cases. This study employed an EUA assay performed in a CLIA-certified laboratory on a venous blood sample, with demonstrated specificity to detect antibodies only to SARS-CoV-2, not to seasonal coronaviruses.

In general, a robust IgG response to SARS-CoV-2 is correlated with the presence of neutralizing antibodies for coronaviruses, but exactly which level of response and which antigen is a reliable predictor for protection has not been established (22). This study only uses the presence of IgG as a measure of exposure. The negative predictive value under any of the reported sensitivity/specificity characteristics is >99.5% for assumed population seroprevalence of <5%. The positive predictive value is 83.6% for an assumed seroprevalence of 5% and 48% for an assumed seroprevalence of 1%. This implies that perhaps even fewer individuals developed antibodies than reported. Alternatively, it is possible that IgG responses are lower in asymptomatic than symptomatic patients and decline over time (23).

In conclusion, based on the data presented in this study, it is unlikely (i) that “herd immunity” can be reached in the foreseeable future and (ii) that asymptomatic spread contributed a substantial number of infections. Taken together, these data suggest that early and rigorous shelter-in-place policies and physical distancing measures are important tools to suppress SARS-CoV-2 infection in the majority of the population.

## MATERIALS AND METHODS

### Enrollment.

Patients enrolled in the study were recruited at nine outpatient clinical sites and two emergency department sites across the state associated with the UNC Health network. Upon arrival for routine care or scheduled visits for enrollment into the study, patients performed a consent procedure that included reviewing recent COVID-19 clinical history using UNC IRB-approved questionnaires. Once consent was obtained, patients provided 5 ml of venous whole blood. This sample was collected by phlebotomists or nurses at each site and analyzed for the presence of SARS-CoV-2 antibody. Results were reported in the UNC medical record, and patients were notified of negative results via an electronic message. For those with a positive result, a physician was responsible for calling the patients and reviewing the results and answering follow-up questions. The ScreenNC protocol was approved by the UNC IRB (UNC IRB 20-0937, NCT04367740). ScreenNC2 samples were sera obtained from UNC patients for indications other than COVID-19. This protocol was also approved by the UNC IRB, which allowed for waiver of HIPAA and consent from these patients (UNC IRB 20-1308).

The comparator cohorts (2019 and 2020) were composed of all patients with any procedure seen in the project testing sites. These include two emergency room sites and multiple outpatient locations. Canceled appointments are excluded. Nineteen departments in the UNC Health System were identified, as some study sites comprise multiple departments. The 2019 cohort covers 15 February 2019 to 15 June 2019, and the 2020 cohort covers 15 February 2020 to 15 June 2020.

### Detection of IgG antibody to SARS-CoV-2.

IgG antibody to SARS-CoV-2 was detected with a microparticle chemiluminescence assay (Abbott Laboratories) on the Abbott Architect i2000SR immunoassay analyzer according to the manufacturer’s protocol under EUA. The Abbott SARS-CoV-2 IgG assay utilizes microparticles coated with SARS-CoV-2 nucleocapsid protein. After washing, bound IgG was detected via addition of anti-human acridinium-labeled second-step antibody. Following a second wash step, pretrigger and trigger solutions were added and a chemiluminescent reaction was detected and reported in relative light units (RLU). The RLU generated is reflective of the amount of antibody bound to the microparticles. The sample RLU was compared to the assay-specific calibrator RLU to generate an index value (S/C). The presence of antibody was reflected by an index value of ≥1.4. Index values of <1.4 were classified as negative for SARS-CoV-2 IgG.

The Abbott SARS-CoV-2 IgG assay was evaluated in-house to assess sensitivity, specificity, and reproducibility. Sensitivity was determined by testing remnant serum samples from patients admitted to UNC Hospitals and confirmed to be infected by RT-PCR testing of nasopharyngeal swabs. Sera collected in the first (*n* = 22), second (*n* = 26), third (*n* = 17), and fourth (*n* = 23) weeks after disease onset were tested. Calculated sensitivities were 31.8%, 80.8%, 100%, and 100%, respectively. This pattern follows the known natural history of IgG antibody development, where titers take 1 to 2 weeks to reach detectable levels after primary infection. Specificity was determined by testing 277 sera. Two hundred thirty-eight of these were collected prior to 1 January 2020 (predating widespread U.S. COVID-19 cases), and 39 were collected after that date. Twenty-three of these 39 patients had nasopharyngeal swabs tested by SARS-CoV-2 RT-PCR and were negative. Additionally, 6 of the 39 samples were from individuals tested for endemic, seasonal coronavirus infection in respiratory secretions and were positive by PCR for the seasonal coronaviruses but not SARS-CoV-2. This group was important to include to establish the specificity of the IgG ELISA for SARS-CoV-2. The remaining 10 sera were from patients with IgM antibodies to cytomegalovirus (CMV) or Epstein-Barr virus (EBV). This set of 39 sera was not “pristine” sera but reflective of the general population in that it included sera with potentially interfering antibodies to other infectious agents. Of the 238 sera collected prior to 1 January 2020, 20 serum samples were from patients containing IgE antibodies to common respiratory allergens, 10 were from individuals with positive antinuclear antibodies, and 9 were from individuals with IgM antibodies to CMV or EBV. Additional sera were obtained from healthy laboratory staff (*n* = 9), pre-solid organ transplant waitlisted patients (*n* = 55), HIV-positive persons (*n* = 6), and a healthy platelet donor cohort (*n* = 129). In total, three serum samples in this set of 277 sera had a positive result. All three were from samples collected prior to 1 January 2020. The estimated specificity of the assay was 98.9%. Intra-assay precision (CV%), determined by performing the 10 replicate determinations of a positive sample, was 1.1%. The same sample was tested seven consecutive days and showed an interassay CV of 0.92%.

### Statistical analysis.

Seroprevalence was estimated using the Rogan and Gladen estimator to adjust for assay sensitivity and specificity. Direct standardization was used to estimate seroprevalence in the UNC Health population. Confidence intervals were obtained using the nonparametric bootstrap percentile method. Specifically, within each stratum of age group, gender, and race, sample seroprevalence was estimated and weighted by the stratum’s proportion in the population. The Rogan-Gladen estimate can be negative, so all estimates of seroprevalence were constrained to be within the interval [0,1]. For comparison, demographic data for patients accessing the screening locations during the time period 15 February 2020 to 15 June 2020 were used.

## References

[1] Zhao J, Yuan Q, Wang H, Liu W, Liao X, Su Y, Wang X, Yuan J, Li T, Li J, Qian S, Hong C, Wang F, Liu Y, Wang Z, He Q, Li Z, He B, Zhang T, Fu Y, Ge S, Liu L, Zhang J, Xia N, Zhang Z 2020 Antibody responses to SARS-CoV-2 in patients of novel coronavirus disease 2019. Clin Infect Dis doi:10.1093/cid/ciaa344. PMC7184337 32221519

[2] To KK, Tsang OT, Leung WS, Tam AR, Wu TC, Lung DC, Yip CC, Cai JP, Chan JM, Chik TS, Lau DP, Choi CY, Chen LL, Chan WM, Chan KH, Ip JD, Ng AC, Poon RW, Luo CT, Cheng VC, Chan JF, Hung IF, Chen Z, Chen H, Yuen KY 2020 Temporal profiles of viral load in posterior oropharyngeal saliva samples and serum antibody responses during infection by SARS-CoV-2: an observational cohort study. Lancet Infect Dis 20:565–574. doi:10.1016/S1473-3099(20)30196-1. 32213337 PMC7158907

[3] Qu J, Wu C, Li X, Zhang G, Jiang Z, Li X, Zhu Q, Liu L 2020 Profile of IgG and IgM antibodies against severe acute respiratory syndrome coronavirus 2 (SARS-CoV-2). Clin Infect Dis doi:10.1093/cid/ciaa489. PMC7197626 32337590

[4] Long QX, Liu BZ, Deng HJ, Wu GC, Deng K, Chen YK, Liao P, Qiu JF, Lin Y, Cai XF, Wang DQ, Hu Y, Ren JH, Tang N, Xu YY, Yu LH, Mo Z, Gong F, Zhang XL, Tian WG, Hu L, Zhang XX, Xiang JL, Du HX, Liu HW, Lang CH, Luo XH, Wu SB, Cui XP, Zhou Z, Zhu MM, Wang J, Xue CJ, Li XF, Wang L, Li ZJ, Wang K, Niu CC, Yang QJ, Tang XJ, Zhang Y, Liu XM, Li JJ, Zhang DC, Zhang F, Liu P, Yuan J, Li Q, Hu JL, Chen J, Huang A-L 2020 Antibody responses to SARS-CoV-2 in patients with COVID-19. Nat Med 26:845–848. doi:10.1038/s41591-020-0897-1. 32350462

[5] Wu F, Zhao S, Yu B, Chen YM, Wang W, Song ZG, Hu Y, Tao ZW, Tian JH, Pei YY, Yuan ML, Zhang YL, Dai FH, Liu Y, Wang QM, Zheng JJ, Xu L, Holmes EC, Zhang YZ 2020 A new coronavirus associated with human respiratory disease in China. Nature 579:265–269. doi:10.1038/s41586-020-2008-3. 32015508 PMC7094943

[6] Wei WE, Li Z, Chiew CJ, Yong SE, Toh MP, Lee VJ 2020 Presymptomatic Ttransmission of SARS-CoV-2 - Singapore, January 23-March 16, 2020. MMWR Morb Mortal Wkly Rep 69:411–415. doi:10.15585/mmwr.mm6914e1. 32271722 PMC7147908

[7] Tong ZD, Tang A, Li KF, Li P, Wang HL, Yi JP, Zhang YL, Yan JB 2020 Potential presymptomatic transmission of SARS-CoV-2, Zhejiang Province, China, 2020. Emerg Infect Dis 26:1052–1054. doi:10.3201/eid2605.200198. 32091386 PMC7181913

[8] Pan X, Chen D, Xia Y, Wu X, Li T, Ou X, Zhou L, Liu J 2020 Asymptomatic cases in a family cluster with SARS-CoV-2 infection. Lancet Infect Dis 20:410–411. doi:10.1016/S1473-3099(20)30114-6. 32087116 PMC7158985

[9] Kim SE, Jeong HS, Yu Y, Shin SU, Kim S, Oh TH, Kim UJ, Kang SJ, Jang HC, Jung SI, Park KH 2020 Viral kinetics of SARS-CoV-2 in asymptomatic carriers and presymptomatic patients. Int J Infect Dis 95:441–443. doi:10.1016/j.ijid.2020.04.083. 32376309 PMC7196533

[10] Hoehl S, Rabenau H, Berger A, Kortenbusch M, Cinatl J, Bojkova D, Behrens P, Boddinghaus B, Gotsch U, Naujoks F, Neumann P, Schork J, Tiarks-Jungk P, Walczok A, Eickmann M, Vehreschild M, Kann G, Wolf T, Gottschalk R, Ciesek S 2020 Evidence of SARS-CoV-2 infection in returning travelers from Wuhan, China. N Engl J Med 382:1278–1280. doi:10.1056/NEJMc2001899. 32069388 PMC7121749

[11] Arons MM, Hatfield KM, Reddy SC, Kimball A, James A, Jacobs JR, Taylor J, Spicer K, Bardossy AC, Oakley LP, Tanwar S, Dyal JW, Harney J, Chisty Z, Bell JM, Methner M, Paul P, Carlson CM, McLaughlin HP, Thornburg N, Tong S, Tamin A, Tao Y, Uehara A, Harcourt J, Clark S, Brostrom-Smith C, Page LC, Kay M, Lewis J, Montgomery P, Stone ND, Clark TA, Honein MA, Duchin JS, Jernigan JA 2020 Presymptomatic SARS-CoV-2 infections and transmission in a skilled nursing facility. N Engl J Med 382:2081–2090. doi:10.1056/NEJMoa2008457. 32329971 PMC7200056

[12] Zhang X, Tan Y, Ling Y, Lu G, Liu F, Yi Z, Jia X, Wu M, Shi B, Xu S, Chen J, Wang W, Chen B, Jiang L, Yu S, Lu J, Wang J, Xu M, Yuan Z, Zhang Q, Zhang X, Zhao G, Wang S, Chen S, Lu H 2020 Viral and host factors related to the clinical outcome of COVID-19. Nature 583:437–440. doi:10.1038/s41586-020-2355-0. 32434211

[13] Wang Z, Yang B, Li Q, Wen L, Zhang R 2020 Clinical features of 69 cases with coronavirus disease 2019 in Wuhan, China. Clin Infect Dis 71:769–777. doi:10.1093/cid/ciaa272. 32176772 PMC7184452

[14] Sood N, Simon P, Ebner P, Eichner D, Reynolds J, Bendavid E, Bhattacharya J 2020 Seroprevalence of SARS-CoV-2-specific antibodies among adults in Los Angeles County, California, on April 10–11, 2020. JAMA 323:2425–2427. doi:10.1001/jama.2020.8279. 32421144 PMC7235907

[15] Xu X, Sun J, Nie S, Li H, Kong Y, Liang M, Hou J, Huang X, Li D, Ma T, Peng J, Gao S, Shao Y, Zhu H, Lau JY, Wang G, Xie C, Jiang L, Huang A, Yang Z, Zhang K, Hou FF 2020 Seroprevalence of immunoglobulin M and G antibodies against SARS-CoV-2 in China. Nat Med 26:1193–1195. doi:10.1038/s41591-020-0949-6. 32504052

[16] Fauver JR, Petrone ME, Hodcroft EB, Shioda K, Ehrlich HY, Watts AG, Vogels CBF, Brito AF, Alpert T, Muyombwe A, Razeq J, Downing R, Cheemarla NR, Wyllie AL, Kalinich CC, Ott IM, Quick J, Loman NJ, Neugebauer KM, Greninger AL, Jerome KR, Roychoudhury P, Xie H, Shrestha L, Huang ML, Pitzer VE, Iwasaki A, Omer SB, Khan K, Bogoch II, Martinello RA, Foxman EF, Landry ML, Neher RA, Ko AI, Grubaugh ND 2020 Coast-to-coast spread of SARS-CoV-2 during the early epidemic in the United States. Cell 181:990–996.e5. doi:10.1016/j.cell.2020.04.021. 32386545 PMC7204677

[17] Bryan A, Pepper G, Wener MH, Fink SL, Morishima C, Chaudhary A, Jerome KR, Mathias PC, Greninger AL 2020 Performance characteristics of the Abbott Architect SARS-CoV-2 IgG assay and seroprevalence in Boise, Idaho. J Clin Microbiol 58:e00941-20. doi:10.1128/JCM.00941-20. 32381641 PMC7383515

[18] Theel ES, Harring J, Hilgart H, Granger D 2020 Performance characteristics of four high-throughput immunoassays for detection of IgG Aantibodies against SARS-CoV-2. J Clin Microbiol 58:e01243-20. doi:10.1128/JCM.01243-20. 32513859 PMC7383546

[19] Rogan WJ, Gladen B 1978 Estimating prevalence from the results of a screening test. Am J Epidemiol 107:71–76. doi:10.1093/oxfordjournals.aje.a112510. 623091

[20] Havers FP, Reed C, Lim T, Montgomery JM, Klena JD, Hall AJ, Fry AM, Cannon DL, Chiang CF, Gibbons A, Krapiunaya I, Morales-Betoulle M, Roguski K, Rasheed MAU, Freeman B, Lester S, Mills L, Carroll DS, Owen SM, Johnson JA, Semenova V, Blackmore C, Blog D, Chai SJ, Dunn A, Hand J, Jain S, Lindquist S, Lynfield R, Pritchard S, Sokol T, Sosa L, Turabelidze G, Watkins SM, Wiesman J, Williams RW, Yendell S, Schiffer J, Thornburg NJ 2020 Seroprevalence of antibodies to SARS-CoV-2 in 10 sites in the United States, March 23-May 12, 2020. JAMA Intern Med doi:10.1001/jamainternmed.2020.4130. PMC12507447 32692365

[21] Okba NMA, Muller MA, Li W, Wang C, GeurtsvanKessel CH, Corman VM, Lamers MM, Sikkema RS, de Bruin E, Chandler FD, Yazdanpanah Y, Le Hingrat Q, Descamps D, Houhou-Fidouh N, Reusken C, Bosch BJ, Drosten C, Koopmans MPG, Haagmans BL 2020 Severe acute respiratory syndrome coronavirus 2-specific antibody responses in coronavirus disease 2019 patients. Emerg Infect Dis 26:1478–1488. doi:10.3201/eid2607.200841. 32267220 PMC7323511

[22] Premkumar L, Segovia-Chumbez B, Jadi R, Martinez DR, Raut R, Markmann A, Cornaby C, Bartelt L, Weiss S, Park Y, Edwards CE, Weimer E, Scherer EM, Rouphael N, Edupuganti S, Weiskopf D, Tse LV, Hou YJ, Margolis D, Sette A, Collins MH, Schmitz J, Baric RS, de Silva AM 2020 The receptor binding domain of the viral spike protein is an immunodominant and highly specific target of antibodies in SARS-CoV-2 patients. Sci Immunol 5:eabc8413. doi:10.1126/sciimmunol.abc8413. 32527802 PMC7292505

[23] Long QX, Tang XJ, Shi QL, Li Q, Deng HJ, Yuan J, Hu JL, Xu W, Zhang Y, Lv FJ, Su K, Zhang F, Gong J, Wu B, Liu XM, Li JJ, Qiu JF, Chen J, Huang AL 2020 Clinical and immunological assessment of asymptomatic SARS-CoV-2 infections. Nat Med 26:1200–1204. doi:10.1038/s41591-020-0965-6. 32555424

[24] Ng D, Goldgof G, Shy B, Levine A, Balcerek J, Bapat SP, Prostko J, Rodgers M, Coller K, Pearce S, Franz S, Du L, Stone M, Pillai S, Sotomayor-Gonzalez A, Servellita V, Sanchez-San Martin C, Granados A, Glasner DR, Han LM, Truong K, Akagi N, Nguyen DN, Neumann N, Qazi D, Hsu E, Gu W, Santos YA, Custer B, Green V, Williamson P, Hills NK, Lu CM, Whitman JD, Stramer S, Wang C, Reyes K, Hakim J, Sujishi K, Alazzeh F, Pharm L, Oon CY, Miller S, Kurtz T, Hackett J, Simmons G, Busch MP, Chiu CY 27 5 2020 SARS-CoV-2 seroprevalence and neutralizing activity in donor and patient blood from the San Francisco Bay Area. medRxiv. doi:10.1101/2020.05.19.20107482. PMC7499171 32943630

[25] Paiva KJ, Grisson RD, Chan PA, Huard RC, Caliendo AM, Lonks JR, King E, Tang EW, Pytel-Parenteau DL, Nam GH, Yakirevich E, Lu S 25 7 2020 Validation and performance comparison of three SARS-CoV-2 antibody assays. J Med Virol. doi:10.1002/jmv.26341. 32710669

